# Editorial: Application of biostatistics and epidemiological methods for cancer research in Sub-Saharan Africa

**DOI:** 10.3389/fpubh.2022.1069098

**Published:** 2022-11-15

**Authors:** Bernard O. Omolo, Samuel O. Manda

**Affiliations:** ^1^Division of Mathematics and Computer Science, University of South Carolina—Upstate, Spartanburg, SC, United States; ^2^School of Public Health, Faculty of Health Sciences, University of the Witwatersrand, Johannesburg, South Africa; ^3^Department of Statistics, University of Pretoria, Pretoria, South Africa

**Keywords:** biostatistics, cancer, disease burden, epidemiological methods, Sub-Saharan Africa

According to the World Health Organization, cancer accounted for about 10 million deaths worldwide in 2020 and has been one of the leading causes of mortality around the world.

Most of the cancer burden is attributed to a few cancer types, including breast, lung, and stomach cancer (1). Previously regarded as the disease of the developed countries, cancer has now become a major public health problem in Sub-Saharan Africa (SSA) and is among the three leading causes of premature death in the region. The cancer burden in the region is expected to increase 2-fold by 2040, with around 1.5 million new cases and 1 million deaths, as a result of unhealthy behavior and lifestyles, aging, and a growing population (1). This Research Topic seeks to gather contributions on the current status of cancer research in SSA, with a special focus on cancer epidemiology and biostatistics.

The Research Topic begins with a scoping review of the burden of the most prevalent types of cancer in SSA (i.e., cervical, breast, and prostate cancer) by Musekiwa et al., followed by a descriptive analysis of trends in prostate cancer incidence between 1997 and 2017 in the Eastern Cape Province of South Africa (Ramaliba et al.), and lastly, the prediction of recurrence and survival of colorectal cancer (CRC) in South Africa, using statistical and machine learning methods (Achilonu et al.).

The scoping review revealed a dearth of cervical, breast and prostate cancer burden studies in most countries in SS, as existing incidence, prevalence and quality of life studies are mainly from Nigeria and South Africa. They recommend that national demographic surveys, which is the common data collection method in these countries, be expanded to include cancer burden studies, especially for cervical, breast and prostate cancer (Musekiwa et al.). Prostate cancer is the most prevalent among men in South Africa (2). Ramaliba et al. report a descriptive analysis of secondary data on prostate cancer from the Eastern Cape Province of South Africa, where they estimated the age-specific and age-standardized incidence rates and noted an increase in these measures of disease burden in that region. It is therefore of public health importance to determine the factors that are associated with this increase in incidence. In this regard, future epidemiologic studies should identify these risk factors in order to inform public health policies on prostate cancer prevention in the region as well as the entire country.

South Africa has the highest incidence of colorectal cancer in SSA among men and women (3). There is an extensive literature on CRC incidence estimation [see, for example (4–6)]. However, little work has been done on CRC prognosis. In this regard, Achilonu et al. developed recurrence and survival models for CRC based on a South Africa population-based data. They employed statistical (logistic regression) and supervised machine learning methods naïve Bayes (NB), random forests (RF), support vector machine (SVM), and artificial neural networks (ANN), where they showed that ANN had the best predictive accuracy. These models can be externally validated in populations with similar demographic profiles in SSA.

An important focus in the future of research in tertiary cancer prevention is in personalized medicine, where tailored treatments for patients are studied at an individual level. Given the genetic diversity of populations in SSA, research approaches on personalized medicine for cancer patients in SSA needs to focus on genomic studies on cancer risk and survival. In addition, since HIV is still prevalent in most SSA countries and studies have shown an association between cancer outcomes and progression and HIV, statistical methods that jointly model HIV, HPV, cancer, and other infectious diseases, would need to be developed in the future.

Bioinformatics methods should play a larger role in identifying common biomarkers and differentially expressed genes in different cancer types. This is more critical in the identification and validation of biomarkers, especially those related to early diagnoses and treatment outcomes. Also, in this era of data science, artificial intelligence tools such as machine learning and deep learning would need to be optimally harnessed for cancer prediction, improved cancer detection in people who have symptoms and optimized cancer treatment.

## Author contributions

BO drafted the editorial. SM proof-read and edited the editorial. Both authors approved the final version for submission.

## Conflict of interest

The authors declare that the research was conducted in the absence of any commercial or financial relationships that could be construed as a potential conflict of interest.

## Publisher's note

All claims expressed in this article are solely those of the authors and do not necessarily represent those of their affiliated organizations, or those of the publisher, the editors and the reviewers. Any product that may be evaluated in this article, or claim that may be made by its manufacturer, is not guaranteed or endorsed by the publisher.
